# High Specificity but Low Sensitivity of Lab-on-a-Disk Technique in Detecting Soil-Transmitted Helminth Eggs among Pre- and School-Aged Children in North-Western Tanzania

**DOI:** 10.3390/tropicalmed9010005

**Published:** 2023-12-21

**Authors:** Humphrey D. Mazigo, Nyanda C. Justine, Jeffer Bhuko, Sarah Rubagumya, Namanya Basinda, Maria M. Zinga, Deodatus Ruganuza, Vyacheslav R. Misko, Matthieu Briet, Filip Legein, Wim De Malsche

**Affiliations:** 1 Department of Medical Parasitology and Entomology, School of Medicine, Catholic University of Health and Allied Sciences, Mwanza P.O. Box 1464, Tanzania; justinenyanda@bugando.ac.tz (N.C.J.); jeffbhuko@bugando.ac.tz (J.B.); mariazinga@bugando.ac.tz (M.M.Z.); ruganuza@bugando.ac.tz (D.R.); 2 Department of Microbiology, Parasitology and Biotechnology, Sokoine University of Agriculture, Chuo Kikuu, Morogoro P.O. Box 3000, Tanzania; sarahleonce94@gmail.com; 3 Department of Community Medicine, School of Public Health, Catholic University of Health and Allied Sciences, Mwanza P.O. Box 1464, Tanzania; n.basinda@bugando.ac.tz; 4 µFlow Group, Department of Bioengineering Sciences, Vrije Universiteit Brussel, 1050 Brussels, Belgium; veaceslav.misco@vub.be (V.R.M.); matthieu.briet@vub.be (M.B.); filip.legein@vub.be (F.L.)

**Keywords:** soil-transmitted helminths, diagnosis, sensitivity, specificity, Lab-on-a-disk, Tanzania

## Abstract

An estimated 1.5 billion people are infected with soil-transmitted helminths (hookworms, *Ascaris lumbricoides* and *Trichuris trichiura*). These infections are targeted for elimination by the World Health Organization (WHO) by 2030, with the main interventions being mass drug administration using albendazole or mebendazole. Tanzania is one of the endemic countries; it has been implementing MDA to school-aged children for more than a decade and the infection prevalence and intensity of infection have declined. Thus, at this point, the monitoring and evaluation of infection prevalence and intensity of infections, and assessing drug efficacy is crucial and requires accurate diagnostic tests. The currently used standard diagnostic test, the Kato–Katz (KK) technique, has several limitations and the WHO is calling for the development and evaluation of new diagnostic tests. The Lab-on-a-disk (LOD) was developed and tested in the endemic areas of north-western Tanzania to evaluate its sensitivity and specificity using KK and the formol-ether concentration technique. The results showed that when using a duplicate KK slide, the LOD had a sensitivity and specificity of 37.2% (95% CI: 30.7–43.9) and 67.3% (95% CI: 63.1–71.3%). Using four KK slides as a standard technique, the overall sensitivity and specificity were 37.7% (95% CI: 33.1–42.6) and 70.7% (95% CI: 65.5–75.6). The LOD attained high specificity but low sensitivity especially in detecting eggs of *Trichuris trichiura*. The LOD technique has potential as a promising diagnostic test, but its sensitivity still requires improvement.

## 1. Introduction

Soil-transmitted helminth (STH) infections are among the most common infections worldwide and affect the poorest and most deprived communities. They are transmitted by eggs present in human faeces, which in turn contaminate soil in areas where sanitation is poor [1]. The main species of STH that infect people are the roundworm (*Ascaris lumbricoides*), whipworm (*Trichuris trichiura*) and hookworm (*Necator americanus* and *Ancylostoma duodenale*) [1,2]. An approximated 1.5 billion people, or 24% of the world’s population, are infected with STH [1]. STH infections are widely distributed in tropical and sub-tropical areas, with the greatest numbers occurring in sub-Saharan Africa, the Americas, China, and East Asia [1]. Pre-school and school-aged children carry the highest burden of STH infections, with available data indicating that over 270 million pre-school-aged children and over 600 million school-aged children worldwide live in areas with intense transmission of these parasites [1,2,3]. In these groups, infections are associated with diarrhea, vomiting, anaemia, and dehydration, leading to growth retardation and decreased cognitive function [1,3]. These adverse health consequences accumulate, resulting in decreased childhood educational performance and increased school absenteeism [1].

In Tanzania, the strategy for control of STH infections, which was implemented more than 15 years ago, is based on mass drug administration of at-risk pre-school and school-aged children with preventive medication, mainly albendazole (ALB) and mebendazole (MEB) [4,5]. According to the World Health Organization (WHO), treatment should be given once a year when the baseline prevalence of STH in the community is over 20% [4,5], and twice a year when the prevalence of STH infections in the community is over 50% [4,5]. The WHO has set a global target to eliminate morbidities due to STH in children by 2030, by regularly treating at least 75% of the children in endemic areas [4,5,6].

Following repeated mass preventive chemotherapies in north-western Tanzania, the epidemiology of STH has changed, with the majority of infected individuals carrying low to moderate infection intensities [7,8,9,10]. The observed changes in intensities of infection present a new challenge to currently used parasitological techniques, especially the Kato–Katz technique, which is used to monitor drug efficacy and the impact of preventive chemotherapy. The Kato–Katz technique is known to have several limitations, including poor sensitivity for low STH infection intensities, hookworm eggs disappear after one hour and slides for hookworms cannot be stored [7]. Thus, continuing to rely on the Kato–Katz technique to evaluate the effects of control programmes for these infections at a time when intensities have significantly declined, can lead to the wrong recording of false negative results in preSAC and SAC harboring low to moderate infection intensities. In this case, the sensitivity of the Kato–Katz technique can be improved by analyzing multiple stool samples collected over three consecutive days, which is costly [7]. Therefore, the WHO call to develop and evaluate new diagnostic techniques is highly valid. The call insists that newly developed techniques should be more sensitive than the existing methods, cheap, robust, fast, simple, easy-to-use, and cost-effective for diagnosing STH infections in the poorest communities of sub-Saharan Africa [11,12].

A recently adopted Lab-on-a-disk (LOD) technique [13] is designed for diagnosis of STH infections, using the floatation–dilution principle and centrifugal forces to concentrate eggs, which can be viewed in one field of view (FOV). An image can then be captured and shared via cloud servers, and analysed by different individuals worldwide at different times [14,15]. The innovation in this technique is the separation of eggs from other faecal matter. It is based on a combined gravitational and centrifugal flotation, with the eggs guided to a packed monolayer enabling quantitation and identification of egg species present in a single FOV, which can be viewed using a microscope eye piece connected to a regular camera. Images of the samples can be saved and uploaded to internet cloud servers [14,15]).

The objective of the current study was to evaluate the performance of the purification method as a part of single-image parasite quantification (SIMPAQ) [13,14]), based on the LOD technique, by quantifying all the STH eggs within the entire separation disk. In the present work, we therefore do not only count the eggs that are accumulated in the FOV, but also those that are visible within the entire disk outside the FOV. The LOD technique has never been evaluated in large field-based studies in endemic areas. The primary aims of the current study were as follows: (i) to assess the prevalence and intensities of STH infections, and (ii) to assess the performance of the LOD technique in diagnosing STH infections among pre-school and school-aged children in north-western Tanzania before treatment, in comparison to traditional parasitological techniques, mainly the Kato–Katz technique and the formol-ether concentration technique. The results show the status of STH infections in pre-school and school-aged children. Furthermore, the study shows the performance of the introduced LOD technique, and its feasibility for use in larger field studies.

## 2. Materials and Methods

### 2.1. Ethics Statement

The current study was approved by the joint Institution Review Board of the Catholic University of Health and Allied Sciences-Bugando and the Bugando Medical Centre (CREC/422/2020), Mwanza, Tanzania. In addition, the study was approved by the National Ethical Committee, Lake Zone Institutional Review Board (MR/53/100/636). The study received permission from the Regional and District Administrative Authorities of Kagera (including Muleba District Council). Before sample collection, parents and guardians received Kiswahili-translated informed consent forms to read and decide if their child/children would take part in the study. Confidentiality of all collected information was maintained. All children who participated in the study received albendazole (400 mg) and PZQ (40 mg/kg) [16], irrespective of their infection status.

### 2.2. Study Setting

The study was implemented at Muleba district, located in the highlands of Lake Victoria in the Kagera region. Muleba district is located at 1°45′ N, 31°40′ E in the north-western part of Tanzania with an area of 10,739 km^2^, of which 62% is covered by Lake Victoria. The district lies at an altitude between 1200 and 1500 m above sea level. The district is highly endemic for STH infections especially in high altitudes areas. A school-based survey reported a hookworm prevalence ranging from 10–59.5%, that of *A. lumbricoides* from 10–16.7% and of *T. trichiura* 7% [10]. Annual MDA using ALB against STH infections is organized within the school environment and focuses on school-aged children. The study involved pre-school and school-aged children purposively selected based on precision mapping results [10], and schools which had a high prevalence of STH infections. The selected schools were Bugasha, Kimbugu, Mazinga, Rwakahoza and Rwazi from the Muleba district.

### 2.3. Study Design, Population, Inclusion, and Exclusion Criteria

A cross-sectional study was implemented among pre-school and school-aged children from five purposively selected primary schools in Muleba district. The schools were selected based on the findings of previous studies, which indicated the schools were highly endemic for STH infections [10,17]. Children were included if they were in the age range of 4–17 years (the age group with high prevalence of STH), enrolled at the selected primary schools, with no history of using ALB in the past 6 months based on the school deworming report, either male or female, and if they had submitted a signed informed consent from their parents/guardians.

### 2.4. Sample Size and Sampling Procedures

The formula for comparing the sensitivity and specificity of diagnostic tests was used [18]. A 95% confidence interval and a power of 0.8 was used to detect a difference of 10% from a sensitivity of 85% (i.e., P_1_ = 85%, P_2_ = 75%) and P_0_ = 77.5%. The minimum sample size needed was 472 participants; when allowing for 10% of participants to be non-respondent or to refuse to participate, the total sample size would be 519 study participants. However, the available laboratory supplies and budget supported a total of 744 school-aged children. For enrolment of children into the study, the study used procedures described elsewhere.

### 2.5. Data Collection

#### 2.5.1. Questionnaire

A face-to-face interview was conducted with each selected child using a pre-tested questionnaire. The questionnaire collected demographic information of the study participants. Finally, the questionnaire produced information about the previous history of anti-helminthic treatment from school records, whether the participant had taken any anti-helminthic drug (prescribed by medical personnel or during a deworming programme at school) or none during their entire life. For young children, the information was collected by interviewing their parents/guardians invited during the sample collection.

#### 2.5.2. Parasitological Screening for Soil-Transmitted Helminth Infections

##### Kato–Katz (KK) Technique

A single stool sample was obtained from each participating child using a labeled stool container with a lid, and processed using the Kato–Katz thick smears technique using a template of 41.7 mg per thick smear. Four KK thick smears were prepared from each collected stool sample, and examined for hookworm eggs within 60 min after preparation by two independent laboratory technicians. The KK smears were examined for eggs of other STH (*A. lumbricoides*, *T. trichiura* and *E. vermicularis*) infections after 24 h. To ensure quality, 20% of all the positive and negative KK thick smears were independently examined by a quality officer blinded to the previous results of the other two laboratory technicians.

##### Formol-Ether Concentration Technique

The formol-ether concentration technique (FECT) is a widely used sedimentation technique for the diagnosis of intestinal protozoa in preserved stool samples [19]. The most commonly used fixatives for stool preservation are either formalin or sodium acetate-acetic acid-formalin (SAF) [19]. The stool samples remaining after preparation of Kato–Katz thick smears were preserved using 10% formalin, and procedures for FECT were carried out at the Catholic University of Health and Allied Sciences parasitology laboratories. Briefly, approximately 1 g of the collected stool samples from each child was preserved in 10 mL of SAF solution containing 10% formaldehyde. It was then roughly broken up and large faecal particles were removed by straining through a sieve into a conical tube [19]. The collected filtrate was mechanically shaken vigorously with 3 mL of diethyl ether followed by centrifugation at 2000–5000 rpm for 2–3 min. The three layers of the supernatant were discarded and the sediments were transferred to a microscopic glass slide. One drop of povidone iodine was added to stain the eggs and microscopic examination was performed [19]. If the final sediment contained a volume of more than 1 mL, the first two steps were repeated, and part of the suspension removed. Subsequently, 2–3 mL of diethyl ether was added to the remaining sediment. The tube was closed with a rubber stopper, shaken vigorously (~30 s), and centrifuged at 2000–5000 rpm for 2–3 min. Eggs of the helminths were examined at low power objective and recorded in the laboratory book.

##### Lab-on-a-Disc Technique

The LOD method uses a floatation solution and centrifugal forces to separate stool and matrix particles from the parasites. This microfluidic tool uses simple and cheap optical components, which are assembled in a robust tool that is portable. The technique has demonstrated excellent sensitivity to detect parasites in animal stool, as well as STH eggs in human samples with low intensity infection during preliminary field tests [14,15]. The technique can handle as much as 1 g of stool.

The standard operating procedures for quantification of parasite eggs in stool samples using the LOD method are as follows:

1. Store fresh stool samples at 4 °C until examination.
2. Take 1 g of faecal sample and dilute in 20 mL of distilled water (DI) in a 50 mL conical centrifugation tube (1:20 dilution ratio).
3. Add a few plastic granules and shake the tube until sample is perfectly homogenised.
4. Filter the mixed sample through stacked polyethylene terephthalate (PET) filters with 200 µm and 20 µm pore sizes to remove granules and particles bigger than 200 µm and smaller than 20 µm.
5. Take the 20 µm filter, turn it over and rinse particles remaining on the surface with 2 mL DI water into one of the wells from a 6-well plate to recover parasite eggs.
6. Transfer rinsed solution to a 2 mL centrifugation vial (Eppendorf) and centrifuge for 3 min at 1500 rpm.
7. After centrifugation, remove the supernatant and resuspend particles in 500 µL of saturated sodium chloride floatation solution (FS) and transfer to a 1 mL syringe.

##### Centrifugation Step (Figure 1)

**Figure 2:**
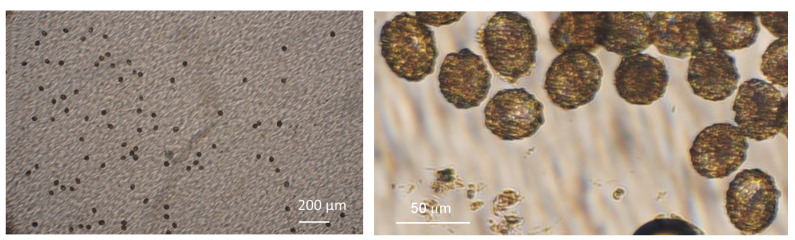
Eggs of *Ascaris lumbricoides* detected in the LOD technique (**left**), a magnified image of *Ascaris lumbricoides* eggs (**right**).

1. Fill the disk with FS.
2. Inject stool sample prepared earlier through the inlet, while the same amount of excessive flotation solution is extracted from the outlet. Injection of the sample solution should be done slowly to avoid particles reaching the imaging zone prematurely. Aim to keep the sample within the first zone (4 mm deep section of the device).
3. After injection of the sample, seal the inlet and outlet with Luer-lock caps.
4. Transfer the disk and fix it in the minicentrifuge. Centrifuge at 5000 rpm for 5 min.
5. Take the device out of the minicentrifuge and screen the disk. All eggs present in the disc are counted (see Figure 2).

### 2.6. Data Management

Data were double entered in a Microsoft Excel 2016 sheet, cleaned, and exported to Stata Version 15 (StataCorp, 2017, Stata statistical software, College Station, TX, USA). Results were recorded for all tests and converted to numerical values (1 = positive and 0 = negative) for analysis. Tables of prevalence and intensity of infection were generated. Disease prevalence was calculated based on the number of positive cases for each diagnostic test. The arithmetic mean egg count was calculated as the average egg count of the four KK smears. For KK technique, arithmetic mean eggs counts were obtained from the counts of four KK smears and multiplied by 24 to obtain the individuals’ number of eggs per gram of faeces. Intensity of infection was categorised according to WHO criteria, defined as low, moderate and heavy intensities of infection [16]. Frequencies/proportions/categorical data were compared using Chi-square (χ^2^) or Fisher’s exact tests and continuous variables were compared using *t*-test. The mean egg counts for STH between sex and age groups were compared using either *t*-test (two groups) or ANOVA (for more than two groups). Sensitivity was determined using KK (two slides and then four slides separately) and FECT as the reference tests. Analysis of sensitivity and specificity of the LOD method followed the procedures described elsewhere [20]. The sensitivity of the LOD method was calculated as the proportion of positive individuals who were correctly identified when compared to the reference tests [20]. Similarly, the specificity of the LOD method was calculated as the percentage of negative individuals correctly identified as negative compared to the reference test [20]. The positive predictive value (PPV) is the proportion of positive test results that are truly positive, and the negative predictive value (NPV) is the proportion of negative test results that are truly negative [21].

## 3. Results

### 3.1. Demographic Information

A total of 744 pre- and school-aged children participated in the study. Of these, 49.5% and 50.5% were female and male respectively. The mean age of participants was 8.7 ± 2.2 years. The age and sex distribution of participants is shown in Table 1 below.

### 3.2. Prevalence and Intensity of Soil-Transmitted Helminths

The overall prevalence of STH infections was 34% (95% CI: 30.6–37.5), 36% (95% CI: 32.6–39.5) and 55.9% (95% CI: 52.3–59.5) using the LOD method, formol-ether concentration technique and Kato–Katz technique, respectively.

Based on two Kato–Katz slides, the prevalence of hookworms, *T. trichiura* and *A. lumbricoides* was 0%, 29% (95% CI: 26.1–32.6) and 38.2% (95% CI: 34.7–41.7), respectively. Based on four Kato–Katz thick slides, the prevalence of hookworms, *T. trichiura* and *A. lumbricoides* was 0%, 35.4% (95% CI: 32.0–38.9) and 41.3% (95% CI: 37.8–44.8), respectively. The arithmetic means for egg intensity based on two KK slides were 58.4 (95% CI: 44.5–72.4) eggs per gram of faeces (epg) and 1054.8 (95% CI: 840.5–1269.1) epg for *T. trichiura* and *A. lumbricoides* respectively. Based on the four Kato–Katz slides, the arithmetic means were 4.7 (95% CI: 3.6–5.8) epg and 82.8 (95% CI: 66.0–99.7) epg for *T. trichiura* and *A. lumbricoides,* respectively.

Based on the formol-ether concentration technique, the prevalence of hookworms, *T. trichiura* and *A. lumbricoides* were 1.9% (95% CI: 0.1–3.2), 19.2% (95% CI: 16.5–22.2) and 26.1% (95% CI: 23.0–29.4), respectively.

Based on LOD, the prevalence of hookworms, *T. trichiura* and *A. lumbricoides* were 0.13% (95% CI: 0.01–0.1), 4.3% (5% CI: 3.1–6.0) and 33.3% (95% CI: 30.0–36.8), respectively.

### 3.3. Sensitivity and Specificity of Lab-on-a-Disc Technique

Considering the two thick smears as a standard Kato–Katz diagnostic test, the overall sensitivity and specificity values (Table 2) were 37.2% (95% CI: 26.3–38.1) and 67.3%. Detailed results are presented in Table 2.

Considering four KK smears as a standard diagnostic test, the sensitivity and specificity of the LOD method were 37.7% (95% CI: 33.1–42.6) and 70.7% (95% CI: 65.5–75.6). Detailed results are presented in Table 3.

Considering the formol-ether concentration technique as a standard diagnostic test, the overall sensitivity and specificity value of LOD were 37.3% (95% CI: 31.5–43.4) and 67.9% (95% CI: 63.5–72). Other results are presented in Table 4.

### 3.4. Estimated Sensitivity, Specificity, Positive and Negative Predictive Values of the LOD Method in Diagnosing Different Species of STH Using KK-Technique and FECT as Standard Tests

The performance of LOD in diagnosing hookworm, *T. trichiura* and *A. lumbricoides* infections using the KK technique as a standard test are presented in Table 5. Overall, the LOD method attained high specificity and negative predictive values in diagnosing *A. lumbricoides* and *T. trichiura* considering KK technique as reference test. Similar results were observation when FECT was used as a standard reference test.

## 4. Discussion

At present, countries endemic for STH infections are working towards achieving the WHO goals of eliminating these infections as a public health problem by 2020–2030 [4], with the main intervention measure being the mass drug administration approach, using albendazole and targeting the most at-risk groups [5]. This means that in the coming years the prevalence and intensity of STH infections will decline and this will be an important challenge to currently used parasitological techniques [22]. Thus, new diagnostic techniques are urgently needed to replace the currently recommended techniques, which have limitations in detecting infections in individuals with a low number of eggs [7,23,24]. In the current study, the performance of the Lab-on-a-disk method was compared with the currently recommended parasitological technique, the KK-technique, and the FECT technique. In general, the LOD technique attained a high specificity and positive predictive values when compared with either standard duplicate or four KK thick smears. Similar results were observed when compared with the FECT technique. The LOD technique achieved lower sensitivity but higher negative predictive values in diagnosing different species of STH included in the study.

Looking at the epidemiology of STH in the current study area, our study confirms that the area remains endemic for these infections and that the majority of the infected pre-school and school-aged children carry low and moderate infection intensities. A recent precision-mapping study noted that Muleba district was among the districts in the north-western side of Lake Victoria which were still endemic for STH infections, despite repeated rounds of mass drug administration [10]. For the country to reach elimination goals [4], the results of the current study and those of other authors call for the need to integrate other intervention measures such as the supply of clean water, improved sanitation within the endemic communities and improved hygiene. Complementing MDA with these measures will help to reduce the transmission forces of these parasites which in turn will impact on the prevalence and intensity of infection.

In relation to the diagnostic performance of the LOD method, there was no difference in its sensitivity when considering duplicate or quadruplicate KK thick smears. The LOD method attained a sensitivity of 37.2% and 37.7% using duplicate or quadruplicate KK slides, respectively. The LOD method attained high specificity using either duplicate or quadruplicate KK slides, at 70.7% and 67.9%, respectively. The negative predictive value (NPV) in both approaches remained <70%, indicating that more than 50% of the STH infected cases in the present study were missed by the test. This was highly pronounced for *T. trichiura* infections. It is important to note that all the children included in this study had low infection intensities for all the STH species included in the study [22,24,25]. In addition, we think that STH eggs, especially those of *T. trichiura*, are lost during the sample preparation procedures. All these factors may partly explain the low sensitivity of the LOD method observed in the current study. The influence of infection intensity on the sensitivity of parasitological techniques has been noted in previous studies [21,22].

Considering individual STH species, the LOD method attained high specificity for *A. lumbricoides*, *T. trichiura* and hookworm using either KK or FECT as a standard diagnostic technique, indicating that the test has a high capacity to correctly identify STH eggs and correctly classify those with no infection. On the other hand, the test had poor sensitivity in diagnosing the STH species, especially *T. trichiura* and hookworms. The prevalence of the latter was very low in the current study population, and this can partly explain the high specificity observed for hookworms.

### Challenges and Limitations

Several challenges were recorded during the evaluation process of the LOD method, including the following: (i) quantification of all STH eggs at the FOV was not possible, because not all eggs enter the FOV, but quantification in the entire disc was possible; (ii) after multiple use of discs, some ended-up broken during centrifugation; (iii) identification of eggs using the provided camera and optical set-up was not always straightforward and this affected the quality of images; (iv) the opening of discs for washing/cleaning after use takes time, this procedure needs to be improved. The observed challenges can be rectified to make the LOD method more user-friendly. On the other hand, the availability of a single stool sample only from each participant and the very low prevalence of hookworms were limitations for the current study. Examination of two to three stool samples is recommended due to the day-to-day variability of eggs [9,26]. This is important for increasing sensitivity of the test especially when working with a population carrying low infection intensities [26]. Infection intensity is used by WHO as an indicator for reaching the elimination stage for STH infection [4,9]. In addition, infection intensity is a measure of morbidities related to STH infections; individuals carrying a heavy intensity of infection are known to have a high degree of severe morbidities [4]. Last but not least, KK technique is not a gold standard to be used for assessing the performance of other diagnostic tests due to its limitations described elsewhere [27], thus the current results need to be interpreted with caution. Despite these challenges and limitations of the LOD method, at this early stage of development the innovation brought by the technique is acknowledged. The ability of the test to capture images of the eggs and save these offline or upload them to the cloud, allowing sharing and remote diagnosis, holds promise. This approach potentially solves the problem of the disappearance of hookworm eggs observed in the KK technique [26]. The LOD method allows laboratory technicians to choose either to focus on processing samples, or taking images and analysing samples, this will have the advantage of the timely release of laboratory results. Future studies are recommended to integrate an image-analysis algorithm.

## 5. Conclusions

The current study assessed the performance of the recently introduced Lab-on-a-disk method, a promising parasitological technique for soil-transmitted helminth (STH) infections. The technique demonstrates low sensitivity for a group of STH infections and for individual STH species when compared with the currently recommended Kato–Katz and formol-ether concentration techniques. Its specificity was satisfactory. Based on the current findings, further research is required to improve the technique with respect to the observed challenges and improve its sensitivity.

## Figures and Tables

**Figure 1:**
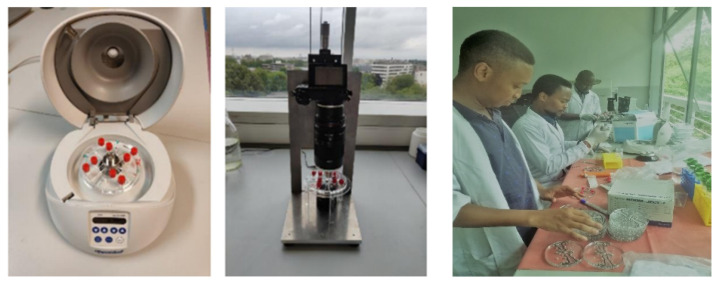
A mini centrifuge with a disk (**left**), camera set-up (**middle**) and the bench workflow of Lab-on-a-disk (**right**) used by permission.

**Table 1:** Age and sex distribution of study participants.

Sex	Age Groups (in Years)		
	4–6	7–10	11–14
Female	53 (44.2%)	242 (52.2%)	73 (45.6%)
Male	67 (55.8%)	222 (47.8%)	87 (54.4%)

**Table 2:** Sensitivity and specificity of the LOD method using two Kato–Katz thick smears as standard diagnostic test.

Diagnostic Test	Sensitivity (95% CI)	Specificity (95% CI)	Positive Predictive Value (95% CI)	Negative Predictive Value (95% CI)
LOD	37.2% (30.7–43.9)	67.3% (63.1–71.3)	32% (26.3–38.1)	72.1% (67.9–76.0)

**Table 3:** Sensitivity and specificity of LOD using four Kato–Katz thick smears as standard diagnostic test.

Diagnostic Test	Sensitivity (95% CI)	Specificity (95% CI)	Positive Predictive Value (95% CI)	Negative Predictive Value (95% CI)
LOD	37.7% (33.1–42.6)	70.7% (65.5–75.6)	62.1% (55.8–68.1)	47.3% (42.8–51.8)

**Table 4:** Sensitivity and specificity of the LOD method using formol-ether concentration as standard diagnostic test.

Diagnostic Test	Sensitivity	Specificity	Positive Predictive Value	Negative Predictive Value
LOD	37.3% (31.5–43.4)	67.9% (63.5–72.0)	39.5% (33.5–45.8)	65.8% (61.4–70.0)

**Table 5:** Estimated Sensitivity, Specificity, Positive and Predictive Values of the LOD method in diagnosing the *T. trichiura* and *A. lumbricoides* infection using KK-technique and FECT as standard diagnostic tests.

STH Species	Considering KK-Technique as Standard Reference Test			
	LOD Method			
	Sensitivity (95% CI)	Specificity (95% CI)	Positive Predictive Value (95% CI)	Negative Predictive Value (95% CI)
*T. trichiura*	7.3% 4.3–11.6	97.0% 95.1–98.3	50% 31.9–68.1	64.5% 60.1–68.7
*A. lumbricoides*	38% 32.4–44.0	69.6% 65.1–73.7	43.5% 37.3–50.0	71.6% 68.2–74.9
**Considering Formol-Ether concentration technique as a reference test**				
Hookworms	0% 0–23	99.9% 99.2–100	0	98.1 96.9–99.0
*T. trichiura*	8.4% 4.4–14.2	96.7% 94.9–98.0	37.5% 21.1–56.3	81.6% 78.6–84.4
*A. lumbricoides*	44.3% 37.2–51.6	70.5% 66.5–74.3	34.7% 28.8–41.0	78.2% 74.3–81.8

## Data Availability

Data are contained within the article.
